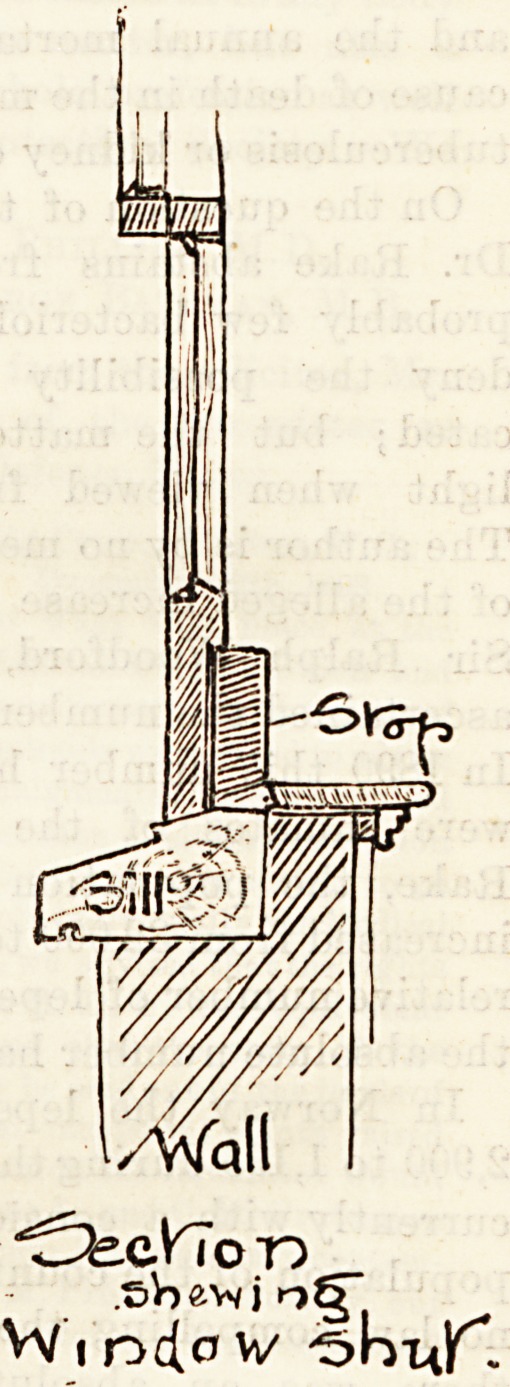# Window Ventilation in Sick Rooms

**Published:** 1893-12-30

**Authors:** 


### PRACTICAL DEPARTMENTS.

### WINDOW VENTILATION IN SICK ROOMS.

The curious popular prejudice against a free inlet of fresh air into sick rooms, or for the matter of that, into ordinary living or sleeping rooms, is wonderfully difficult to overcome, and the process is not rendered easier by the shape and construction of the windows to be found in too many houses, even in these days, when we should expect to find the important question of ventilation more generally understood. It is true that in modern hospitals and similar institutions the old faults are practically eliminated, and in recently-erected buildings of this class it is the rule and not the exception to find ample facilities for efficient ventilation. But while the nurse's difficulties in this respect are reduced to a minimum in the ward of the modern hospital, private nurses have constantly to battle, not only with the prejudices of their patients, but with doors and windows which refuse to lend themselves to well-meant efforts to bring about an improved atmospheric condition. In old-fashioned country houses sash windows are unknown, and with casement windows, innocent of any special arrangement for ventilation at the top, it is a difficult, not to say impossible, feat to so arrange matters that the necessary fresh current of outside air can be freely admitted without, in cold and windy weather, causing draughts and general discomfort to the occupants of the room. The simplest plan with casement windows is to have a small pane at the top, made to open inwards like a hospital sash.

The sash window presents fewer difficulties; indeed, a sash window opened slightly at the top leaves little to be desired as a method of ventilation. But there are many days in this climate when even a few inches thus open at the top may prove too much for comfort, as in cases where the door is immediately opposite the window, when an unpleasant draught is the result.

The sketch we give above illustrates a good and simple plan for obviating this by placing a board fitted to the size and shape of the sill, beneath the lower sash. The fresh air is thus admitted in an upward direction, mingles with the warm air of the room, and is not made unpleasantly apparent in the guise of a down-coming draught. This plan is largely carried out in new buildings, by the lower sash and sill being made sufficiently deep to allow of air being freely admitted in this manner.

Where we find Venetian blinds it is easy enough to give the in-coming air the desired upward direction. "It is only necessary to open the top sash, pull the Venetian blind down in front of the opening, and place the louvres so that they give the entering air an upward direction." Or, where Venetian blinds are not, a board placed in a slanting position can be used for the same purpose and with equal effect.

These are simple methods of making the best of existing arrangements, but they are as effectual as more elaborate means of ventilation, and will frequently be called into requisition by private and district nurses.

## Figures and Tables

**Figure f1:**
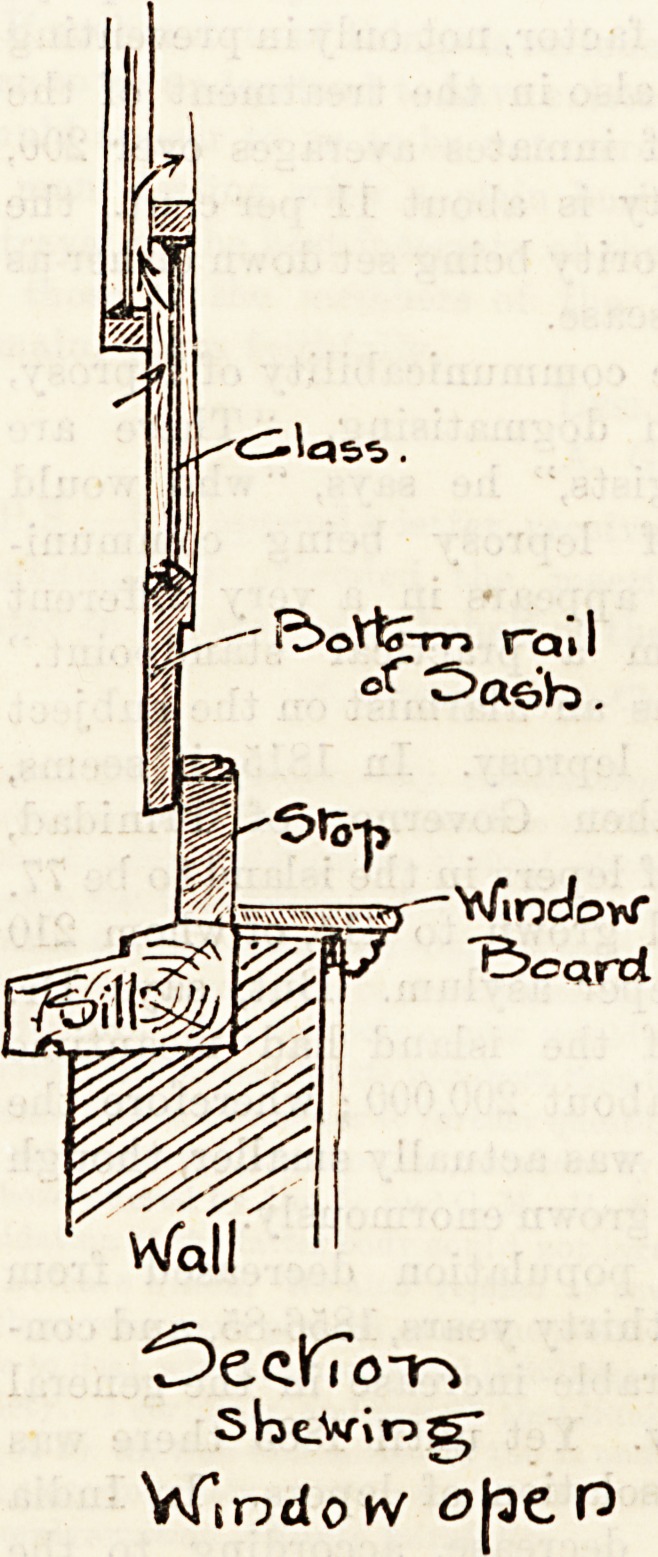


**Figure f2:**